# Intern as Patient: A Patient Experience Simulation to Cultivate Empathy in Emergency Medicine Residents

**DOI:** 10.5811/westjem.2017.11.35198

**Published:** 2017-12-14

**Authors:** Sara W. Nelson, Carl A. Germann, Casey Z. MacVane, Rebecca B. Bloch, Timothy S. Fallon, Tania D. Strout

**Affiliations:** Maine Medical Center, Department of Emergency Medicine, Portland, Maine

## Abstract

**Introduction:**

Prior work links empathy and positive physician-patient relationships to improved healthcare outcomes. The objective of this study was to analyze a patient experience simulation for emergency medicine (EM) interns as a way to teach empathy and conscientious patient care.

**Methods:**

We conducted a qualitative descriptive study on an in situ, patient experience simulation held during EM residency orientation. Half the interns were patients brought into the emergency department (ED) by ambulance and half were family members. Interns then took part in focus groups that discussed the experience. Data collected during these focus groups were coded by two investigators using a grounded theory approach and constant comparative methodology.

**Results:**

We identified 10 major themes and 28 subthemes in the resulting qualitative data. Themes were in three broad categories: the experience as a patient or family member in the ED; application to current clinical practice; and evaluation of the exercise itself. Interns experienced firsthand the physical discomfort, emotional stress and confusion patients and families endure during the ED care process. They reflected on lessons learned, including the importance of good communication skills, frequent updates on care and timing, and being responsive to the needs and concerns of patients and families. All interns felt this was a valuable orientation experience.

**Conclusion:**

Conducting a patient experience simulation may be a practical and effective way to develop empathy in EM resident physicians. Additional research evaluating the effect of participation in the simulation over a longer time period and assessing the effects on residents’ actual clinical care is warranted.

## INTRODUCTION

Empathy is an important trait for compassionate and effective physicians. A number of studies link positive physician-patient relationships and physician empathy to improved healthcare outcomes,^1^,^2^,^3^ enhanced patient adherence with recommendations,^4^ and improved patient satisfaction.^4^,^5^ A systematic review by Stewart found that empathy and support, clear information from doctor to patient, and shared decision-making were associated with improved outcomes and patient adherence.^2^ Additionally, studies show that physician empathy is beneficial to the provider as it is associated with lower rates of physician burnout^6^ and fewer medical-legal risks.^7^

In the medical literature, empathy is defined in several ways. Mercer and Reynolds define physician empathy as the ability “(a) [to] understand the patient’s situation, perspective and feelings (and their attached meanings); (b) to communicate that understanding and check its accuracy; and, (c) to act on that understanding with the patient in a helpful (therapeutic) way.”^8^ Hojat defines empathy as the “cognitive attribute that involves an ability to understand the patient’s inner experience and perspective and a capability to communicate this understanding.”^9^ A simpler definition comes from the Oxford-English dictionary where empathy is “the ability to understand and share the feelings of another.”^10^

Empathy is an important topic in undergraduate and graduate medical education. In recent decades there has been an emphasis on teaching and evaluating psychosocial skills such as empathy and concern, as well as on communication skills and shared decision-making. The Association of American Medical Colleges (AAMC) discusses empathy in its Learning Objectives for Medical School Education stating, “physicians must be compassionate and empathetic in caring for patients.”^11^ The United States Medical Licensing Examination (USMLE) includes an assessment of communication skills for medical students and the Accreditation Council for Graduate Medical Education (ACGME) includes interpersonal and communication skills, professionalism and patient-centered communication in its milestones for many specialties.^12^

In an attempt to teach empathy and to introduce aspects of the patient and family experience to residents, our emergency medicine (EM) residency holds a patient experience simulation for the incoming intern resident class. The purpose of this study was to explore residents’ perceptions of the simulation, with particular attention to the development of empathy.

## METHODS

We conducted a qualitative descriptive study and used focus groups as a means of generating study data. In July 2016, all 10 EM interns at the study institution participated in a patient experience simulation during their first week of residency orientation. The study institution is a Level I trauma center and the emergency department (ED) cares for approximately 80,000 patients annually. The EM residency is three years in length, with a total of 28 residents.

This was an in situ simulation, held in the ED. Half of the interns had the role of “patients” injured in a motor vehicle accident. These patients were brought into the ED by a professional ambulance service on a backboard with a cervical collar (C-collar) and were evaluated in the trauma bays by a senior EM resident, a medical student and two nurses. The other half of the interns had the role of “family member” and went through the process of arriving at the ED and locating their family member after an accident. The complete simulation lasted three hours and included a full trauma assessment (including the use of moulage clothing that could be cut off), continuing ED care, time in the family waiting room, transport to radiology, splint placement, the need for pain medications, the need to use the bathroom, the use of crutches and receiving discharge instructions. While efforts were made to make the experience realistic, participants were not irradiated with radiographs, did not have intravenous (IV) lines placed and received no medications. After the exercise, the interns completed a brief evaluation form on their experience and a standard hospital patient satisfaction survey. To conclude the experience, there was a debriefing moderated by faculty members.

**Population Health Research Capsule**

What do we already know about this issue?

Research has linked empathy and positive physician-patient relationships to improved healthcare outcomes. A variety of educational interventions have been used to teach and evaluate empathy and psychosocial skills in medical education. Despite these efforts, studies show that empathy may decrease during medical training, which may have direct consequences on patient care and physician well-being.

What was the research question?

Would participation in a patient experience simulation by EM interns during orientation help them to develop empathy for patients and their family members?

What was the major finding of the study?

Conducting a patient experience simulation may be a practical and effective way to nurture the development of empathy in EM residents and foster their responsiveness to patients’ needs and concerns.

How does this improve population health?

Implementation of an intervention and curricular tool to cultivate empathy in resident physicians would help improve patient-physician relationships and prevent empathy degradation in medical education. In addition to its value for EM residencies, patient experience simulations could also be developed for interns and students in other specialties.

All participants in the simulation were invited for a follow-up focus group held during a scheduled research meeting when the majority of residents were available. Focus groups were chosen for data collection in order to encourage cross-talk and idea generation between the residents. Residents were informed that their participation was voluntary, their responses were confidential, and their decision to participate would not affect their job or standing in the residency program. Eight of the 10 interns were available to participate in the 60- minute focus group.

Residents were interviewed by two investigator moderators [SN, CM] using a semi-structured interview guide. There were 28 questions organized into themes highlighting patient needs, staff communication, challenges with the experience, empathy, and future simulations. (See appendix for Focus Group Moderator Guide.) The focus group was audio-recorded and the recording was transcribed by a CITI-certified transcriptionist. All resident responses were de-identified, kept strictly confidential, and reviewed only by members of the research team. The study was deemed exempt by the Maine Medical Center Institutional Review Board.

### Data Analysis

Using a constant comparative method and a grounded theory approach,^13^ two investigator coders [SN and CG] identified themes in the resulting qualitative data with phrases as units of analysis. These qualitative methods were appropriate for early exploration of a phenomenon such as this, when the goal was to gain an understanding of participants’ views and experiences.^14^ The transcripts were first read, generally, to provide an overall impression of the major topics discussed. They were then read line-by-line by the two independent coders. Themes and subthemes were formulated through an inductive process. The coders then met in person to develop a consensus framework. The two investigators then independently recoded the transcripts with this new coding schema, using the process of constant comparative analysis, until new themes no longer were identified in the data (saturation). They then met again to develop a final coding schema.

The investigators used this final framework to code the transcripts for a third time. The investigators adjusted the coding by consensus, following each round of coding in an iterative process. The agreement between coders was excellent on the third round of coding with all unit phrases fitting within the coding scheme. After the final coding, illustrative quotes were selected. To improve readability, we corrected quotes for grammatical errors.

## RESULTS

Of the eight interns interviewed, three were in the role of patient and five were in the role of family member. Three were female, five were male, and all were between 26 and 33 years old. All participants were EM interns and none had any prior medical residency training.

Ten major themes and 28 subthemes were identified in the participant comments. The themes fell into three broad categories: the experience as a patient or family member in the ED; the application to current clinical practice; and the evaluation of the exercise itself. See table for themes and illustrative comments.

### Experience as a Patient or Family Member in the ED

The intern participants discussed the physical discomfort and the emotional stress of the ED process. They described how the process felt unfamiliar and confusing and they speculated about how patients and families with little medical literacy would feel. Finally they discussed examples of poor communication between ED staff and patients and their families.

The physical discomfort was a surprise to several of the participants. One participant noted, “I did not understand or appreciate that … those C-collars are awful.” Another participant noted the discomfort of the monitoring stickers, commenting. “Really painful and awkward.”

More impactful than the physical stress was the emotional stress for both patients and family members. The interns commented about feeling vulnerable and feeling anxious. A family member said “you have very little control and you always want answers.” A patient commented, “Like when they take off your clothing to do the secondary survey… is very like emotionally uncomfortable.” In addition, there were comments about the lack of accommodations for patients and for families and the lack of privacy. There were comments about waiting for information and updates. “[There was a] prolonged wait time for just finding out what the next step was.” Finally, participants expressed feeling burdensome to staff. One family member said, “You don’t want to be the annoying person that is raising your hand all the time.”

The third theme was that patients and family members were unfamiliar with the overall process of ED care. The comments were divided among disorientation to surroundings and being unclear about the plan of care. A patient who arrived on a backboard said, “I got out of the ambulance. I had no idea where I was … ‘cause all you could see was the ceiling.” Family members were also confused and unsettled to the surroundings. The process of medical care and the flow through the ED was also unclear to participants.

The last theme identified was how poor communication affected their experience. Both patients and family members raised examples of lack of communication as well as of poor communication skills. While most comments reflected a need for more communication, at times the attitudes of staff were felt to be poor.

### Application to Current Clinical Practice

The focus groups were held four months into intern year. Three major themes and eight subthemes were formulated about what residents learned or experienced during the exercise and will try to apply to their practice. The residents discussed the importance of good communication skills, the importance of good communication content including setting expectations on the process and timing, and the importance of overall good patient care, which included patient advocacy and being responsive to patients’ needs and concerns.

Residents described examples of both good and bad communication skills. There were comments about the importance of making eye contact when speaking to a patient, especially patients lying on their backs with C-collars in place. A patient said, “If you’re in a C-collar you can’t see anything, so you gotta [sic] lean right over them.” Residents also described the impact of simply having positive interactions when communicating.

Mentioned often was the importance of good communication content. Participants discussed setting expectations on ED process for patients and family members. One resident commented, “In terms of laying out the visit … I feel is really valuable.” In addition, residents discussed the importance of setting expectations about timing for the visit. One resident noted, “I think setting expectations early on is really important and overestimating time waits is really important.” Providing frequent updates about care was also formulated as an important subtheme.

A final theme was the importance of good patient care, which pertained to honoring the relationship and trust between caretaker and patient and family members. Participants discussed the need to be a patient advocate. Residents also discussed the importance of being responsive to the needs and concerns of patients and families as representing good patient care. One resident commented it was important to “normalize the experience” and “acknowledge people’s discomfort.” Finally, residents felt it was important to be cognizant of time with regard to length of stay and time between updates. One resident remarked, “It changed how I think about my patients … I realize when something is taking forever… I go in and say ‘I am sorry that this taking so long.’”

### Evaluation of the Exercise Itself

The interns were extremely positive about the simulation and commented how useful the act of discussing the exercise in the focus group was to reinforce the experiences and concepts they had learned. In the discussion, formulated themes were the strengths of the exercise, the limitations of the exercise and future directions.

One of the strengths of the exercise was its realism. One resident commented, “I thought that was super valuable to know what it’s like to be on that backboard, to be looking at the ceiling and have paramedics ask questions.” Some interns also felt a benefit of the exercise was the role modeling, especially by the senior EM resident who was caring for them.

Participants mentioned several limitations of the exercise. The patients were prompted to be in pain and to ask for pain medications. This part of the simulation lacked credibility for the participants. One patient said, “We weren’t experiencing pain. It was really hard to advocate for ourselves.” The other limitation of the exercise was that interns had prior exposure to the ED and prior medical knowledge. Some felt that this might have colored their simulated experience.

Comments about future directions for the simulation included new ideas and general support for the exercise. Interns valued the feelings of disorientation and anxiety. They also liked the timing during intern orientation as a way to add to the power of the simulation. One resident commented, “I think doing it early before people know the staff and know the location [is important].”

There were comments about new ideas for the exercise. “Maybe take away the cell phones of the patients.” Another suggestion was to establish IV access on the patients. One suggestion for improvement was to clarify the relationship between the participant patients and family members and to be more specific when coaching the patient history.

When asked whether the simulation would influence how the interns care for patients and how they empathized there was a resounding “Yes.” One participant commented that the focus group itself was useful, “I feel this is a useful thing … [to] reflect what was it like to be a family member or what it was like to be a patient, cause when you start … working like 12 days in a row, you get tired and you … kind of forget.” When asked about the timing of the simulation during orientation as well as the length of the exercise, there was also general support. When asked if the orientation should be held for interns next year, there was again a resounding “Yes.”

## DISCUSSION

Empathy is a vital topic in undergraduate and graduate medical training. In the field of EM, Patient-Centered Communication and Professionalism are two of the ACGME Milestones.^12^ In addition, empathy, humanism and professional values are discussed in the Emergency Medicine Doctrine of Professionalism.^15^ A growing body of literature has shown that empathy and good communication directly enhance the therapeutic efficacy of physicians^16^ and that training in these psychosocial and communication skills is effective.^17^ In a recent review of empathy training in medical education by Kelm, the majority of interventions were aimed at medical students.^18^ At the same time, several studies show that provider empathy may decline during medical school and residency.^18^

Our study builds on work by MetroHealth Medical Center in Cleveland, Ohio, which conducted a similar patient-experience simulation for its EM residents.^19^ At the University of Florida Health Science Center, EM interns experienced the ED process from registration to triage to seeing the hospital bill through a clinical scenario (e.g. sore throat, back pain, headache).^20^ At the Long Beach Memorial Medical Center family medicine program, residents were admitted overnight to the hospital.^21^ Our study adds to this body of literature by using a qualitative approach to understand the experience and the lessons learned from interns four months after being treated as patients and as family members in a busy ED.

Our study proposes a feasible intervention and curricular tool to cultivate empathy in resident physicians. Participants experienced firsthand many of the challenges of being patients and family members in the ED. They experienced aspects of care including the discomfort of wearing a cervical collar, the emotional stress of waiting for care updates, and the disorientation of the ED setting. The interns felt the simulation taught and reinforced several important aspects of being a conscientious and caring provider. They described good communication both in terms of content – setting expectations on the process, setting expectations on the timing and providing updates, and in terms of delivery – making eye contact, having positive interactions. They also discussed good patient care in terms of being a patient advocate, being responsive to needs and concerns, and being cognizant of timelines and waits. Several times the interns noted that the process of remembering and discussing the simulation was a valuable exercise to reinforce the aspects of patient care and empathy that they had learned.

A patient experience simulation where learners are placed directly into the environment in which they will be working is a transferable learning experience for residents and medical students in many subspecialties. This seems particularly important for interns who are starting residency and making the distinct transition from medical school into a service role as hospital housestaff. Our patient simulation exercise could be used in EM residencies across the country, and similar simulations could also be developed for interns and students in other specialties. We plan to publish the details of this patient experience simulation so that other educators and residencies may use it in their curriculum.

## LIMITATIONS

There are several limitations to be considered when interpreting the findings of this study. First, this work included a small number of participants (8 of 10 interns) from a single academic center. There were also challenges to the fidelity of the simulation exercise for the intern patients. As the participants were not actually injured, it was difficult to simulate the pain experience when they were instructed to request pain medication. In addition, ED staff were not blinded to the exercise: EMS, registration, nursing and ED technicians all knew that the interns were participating in a simulation experience.

Finally, the investigators facilitating the focus groups were the same faculty who organized and ran the patient experience simulation. The investigators discussed their own reflexivity prior to the focus groups and were given an explicit facilitator guide. During the focus groups, ground rules were reviewed and confidentiality was emphasized. Despite this, it is possible that the interns were trying to please the faculty when answering questions. Having an external moderator run the focus group may have allowed interns to be more objective.

## CONCLUSION

Empathy, good patient communication, professionalism and humanism are important skills in medicine that not only aid in cultivating the doctor-patient relationship but also improve patient outcomes and physician work satisfaction. Unfortunately, the natural empathy that providers have when they start medical school may wane with the rigors of medical training. To combat this, residency programs must find innovative ways to teach, reinforce and evaluate provider empathy and communication skills.

Findings from this study suggest that conducting a patient experience simulation may be a practical and effective way to nurture the development of empathy in EM residents. Additional research is warranted to evaluate the effect of participation in such experiences over a longer time period and to assess the effects on residents’ actual interactions with patients and families while delivering care.

## Supplementary Information



## Figures and Tables

**Table:** Themes and subthemes with illustrative comments

Theme	Exemplar comment
Physical discomfort	
C-collar and back-board	I did not understand or appreciate that those C-collars are awful. If I was at all not in my right state of mind, and I was like at all trying to take charge of my healthcare, I probably would have actually considered taking that thing off. It was really, really uncomfortable and painful and digging into my ears
Other	The stickers on your chest, pulling those off, I was missing a patch of chest hair for months. Really painful and awkward.
Emotional stress	
Long wait times	I mean I knew this was a simulation so I knew that somebody would come and get me eventually but I was thinking if I was just some person waiting for my family member, I would see people walk by and it was a long time and if I was probably really here I probably would have looked for somebody and been like “hey do you remember that I’m here?”
Lack of privacy	Patients can hear what you are saying. They don’t care about your Facebook because they are having one of the worst days of their lives.
Feeling vulnerable	When they take off your clothing to do the secondary survey ... I am like realizing that that has to be a very vulnerable feeling to be there with a sheet over and you and people you don’t even know rolling you ... that would be something I now acknowledge is very emotionally uncomfortable.
Feeling of anxiety	I don’t think I have ever really internalized that and thought about how anxiety provoking it could be for those patients. I provide them the verbal reassurance that we are there to take care of you but I don’t think I’ve ever really thought about how stressful that could be to like ask could I please use the bathroom.
Lack of accommodation (for patients and family)	We were in a hallway bed and I always felt like I was in the way. People were pushing beds up and down that hall in that space on the B-side and then there wasn’t a chair or anything to sit and I was just kind of dodging everybody constantly.
Feeling burdensome to staff	I felt very self-conscious and that I was asking for something really annoying and taking their time, but the nurse didn’t show any sign in her face or tone of voice whatsoever that it was annoying request and was totally professional about it.
Other	Feeling very frustrated and feeling very isolated.
Poor communication	
Lack of communication	It’s very anxiety provoking especially when you are in that room initially waiting for information.
Poor communication skills	I never thought that there would be so much abrasiveness associated with being a family member at a hospital even when you are not peppering the staff with questions or raising your hands to ask.
Other	Having no concept of who was communicating with me.
Unfamiliar with overall process	
Unclear about plan of care	There were a lot of things happening at the same time and I also didn’t see anybody, so I feel it was just, like there were a lot of things happening at once.
Disorientated to surroundings	I remember not being told where I was going and having no idea where I was.
Unclear expectations of time	There were certain times that we had no idea how long the current activity was going to last and what was up next.
Good communication content	
Providing updates on care	I guess it made me more cognizant of trying to find family members or update them when people tell me that their family members are coming.
Setting expectations on the process	Lying out the visit, which is something I hadn’t developed as a first year what actually I can do, what like a typical plan for a patient looks like. But now letting people know about that I feel is really valuable.
Setting expectations on timing	I think setting expectations early on is really important and overestimating time waits is really important.
Theme	Exemplar comment
Good communication skills	
Positive interactions with staff	They both have very warm personalities, which helped. Our questions were very well answered. Our concerns were addressed by the attendings. I did notice that your time with them is very, very short. Which is probably realistic, we probably spent less than two minutes with each of them combined ... so most of your time is with the nurses ... which you don’t always necessarily realize as a resident or as an attending -- you are not the most important person in the room, it is typically the nurse, and I think this highlighted that in many ways.
Making eye contact	Every time I have a patient in a C-collar I lean over and make sure that they can see my face and explain my role and who I am and all of the people that are around.
Other	Just reiterating good communication.
Good patient care	
Patient advocacy	Acknowledging people’s discomfort… that this is a strange experience and be sure to ask questions of how I can make that better for them, just little things.
Responsive to needs and concerns	Just normalized for them if they haven’t eaten to speak up and don’t feel bad about it, don’t feel self-conscious about like bugging us and asking for help if they need anything just because that was something more comfortable from my experience I think to let them know that it’s okay to do.
Being cognizant of time	It changed how I think about my patients when it comes to time because I was sitting around waiting for so long. So I realize when something is taking forever, I don’t think that I did it previous to this experience, but I go in and say “I am sorry that this is taking so long.”
Limitations of the exercise	
Difficult to simulate pain	I feel like pain is such a subjective thing and like we weren’t experiencing pain it was really hard to advocate for ourselves.
Prior knowledge or experience	I feel like it was biased though because we knew what that was because I’ve done it on the other side. It made sense so even if he wasn’t explaining it, I still understood.
Strengths of the exercise	
Realistic exercise	I thought that was super valuable to know what it’s like to be on that backboard to be looking at the ceiling and have paramedics ask questions.
Role modeling	I just remember the whole time being impressed by [senior resident] really. Being like, I hope I can do that one day.
Future directions	
Keep disorientation and anxiety	I think doing it early before people know the staff and know the location.
New ideas	I would tell us whether or not you want us to give a true history or not, just to clarify what we are supposed to be saying when people ask questions. I would definitely have us like keep wearing those fake clothes. That was really helpful. I think having it as real as possible, so if you want to place IVs, that would have been not outside the realm of possibility. I mean you put the cast on for Pete’s sake; you might as well do everything else real.
General support for exercise	Yes (response to: “Do you guys think that this simulation influenced how you care for patients?”) Yes (response to: “So should we do this next year with the interns?”)
