# Supplementary material for: Intern as Patient: A Patient Experience Simulation to Cultivate Empathy in Emergency Medicine Residents
Source: West J Emerg Med. 2017 Dec 14;19(1):41–8. doi: 10.5811/westjem.2017.11.35198 (PMC5785200; doi:10.5811/westjem.2017.11.35198)
Supplement: Supplementary file 1 [file wjem-19-41-s001.doc]

**Patient Experience Simulation MODERATOR’S GUIDE**

**Participants: PGY 1 EM Residents**

**RESEARCH PURPOSE:**

The purpose of this study is to explore the experience of residents who participated in a patient experience simulation during their intern orientation. We are interested in what aspects of the experience they appreciated and how the experience has affected their practice as physicians and their empathy for patients and for visitors. A qualitative descriptive study design will be utilized, which will allow for a formal collection of relevant themes. A focus group methodology will be utilized to capture the data.

**SUPPLIES**

- Name cards and/or tags
- Digital tape recorders (2)
- Batteries
- “Do not disturb” sign
- Handouts: extra consent forms, ground rules, demographic forms
- Snacks (not crunchy) and beverages

**HELPFUL HINTS**

Preparation Activities

- Collect informed consents/assents ahead of time (need 10-12) to ensure group size of 8-10
- Collect all supplies

Setup

- Ensure that you won’t be interrupted
  - Hang sign on door
  - Check out room schedule
- Put pad under recorder
- Plan for equipment failure

Other

- Stop and start the tapes at different times
- Anticipate pitfalls:
  - “No shows”
  - Groupthink
  - Dominance by a group member
  - Questions that fall flat

Exemplary probing questions to facilitate discussion

- “Can you tell me more about that?”
- “How so?”
- “Can you share an example?”
- “How do others feel?”
- “Did others have a similar experience?”
- “Did anyone remember something different?”
- “So I think you said, XXXX, is that right?”

**WELCOME**

**Purpose**

Welcome. Thanks for coming. I am _____________________ and this is __________________ (recorder’s name). We are here today to learn from you. At the start of your intern year we asked that you participate in a patient experience simulation. Half of you were put into the role of a patient that had been injured in a motor vehicle accident and were transported to the MMC ED by ambulance on a backboard. The other half of you played the role of family member and went through the process of entering the ED, registration and supporting your family through the ED care process. The exercise lasted for approximately three-four hours and after the exercise we did discussed the simulation to obtain your impressions and thoughts. We also had you fill out a standard hospital patient satisfaction survey.

It is now four months (or 16 months) later and we would like to know what you appreciated, liked and didn’t like about the patient experience simulation. We would also like to explore how, if at all, the experience changed your understanding of the ED patient and family experience and if it changed how you care for your patients.

We want to hear what you think and feel about the simulation. We appreciate that you have agreed to participate in this discussion because we value your opinions. Everybody’s input is important, so please feel free to discuss what you think with us. There are no right or wrong answers; we want your opinions. The information you give us will help us to create additional learning experiences for residents.

**Disclosures**

We are going to be together for the next hour. We are recording this session because everything you say is important and we could not possibly remember it all or write it all down, but no one outside this project will ever know who said what. The only exception is if you say you have thoughts about hurting yourself or others or you tell us about someone who has made threats about hurting themselves or others. __________________ (recorder) is here to help record; she will also be taking notes, and will not be joining us in the discussion. We also won’t use your names in our reports. Just use your first name.

**Ground Rules**

There are no right or wrong answers to any of the questions, every opinion is important. We expect that some of you will have different points of view from each other. Please feel free to share what you are thinking. It is OK to disagree with something that someone else has said but we need to do it respectfully. You do not have to answer any of the questions if they make you feel uncomfortable. From time to time I may call on one of you but that’s only to make sure you get a chance to speak. Just shake your head “no” if you do not have something to say. That will be OK.

Let’s establish the ground rules for this discussion. What do you think about these? (pass out ground rules).

- Only one person talks at a time.
- Just use your first name. State your name before you answer a question.
- Be polite to each other…try not to interrupt.
- Keep on the subject.
- It is OK to eat during our discussion.

Are there other ground rules we need? Are there any questions at this point about how we’ll be doing this?

**PARTICIPANT INTRODUCTIONS**

**General Introduction:**

(The main purposes are to start the warm-up, match each person’s name and voice on the tape, get each person to speak once, and get the group warmed up and comfortable).

Let’s get started by going around the group one at a time:

- First names
- Residency class
- What role you played in the simulation (patient or family member)

**INTRODUCTORY QUESTION**

- What aspects of the patient experience simulation do you remember most?

**CHALLENGE QUESTIONS**

- What were the most challenging aspects of being a patient?
- What were the most challenging aspects of being a family member?
- What parts of the pre-hospital/ED process did you find physically challenging/uncomfortable?
- What parts of the pre-hospital/ED process did you find emotionally challenging/uncomfortable?

**STAFF QUESTIONS**

- What did a staff member do for you that was helpful?
- What did a staff member do for you that was kind?

**NEEDS QUESTIONS**

- How was your pain managed?
- How were your bodily needs handled?
  - Hunger? Thirst? Temperature? Bathroom needs?
- Was there anything that a staff member did that did not represent good patient care?

**COMMUNICATION QUESTIONS**

- What parts of the pre-hospital/ED process did you find confusing?
  - Did you understand the next steps in your care process?
- How were your expectations of time managed?
- Was there any part of the process that could have been made easier for the patient?
- Was there any part of the process that could have been made easier for the family member?
- Were there times when staff communication could have been improved?
- Are there ways that staff communicated well with you during the process?

**CURRENT PRACTICE AND EMPATHY QUESTIONS**

- What aspects of the patient experience simulation have affected your care of patients? How?
- What aspects of the patient experience simulation have affected your patient empathy? How?
- Are there things that you do to care for your patients and their family members/support that are influenced by the simulation? What?

**FUTURE SIMULATION QUESTIONS**

- What aspects of the patient simulation experience should we keep?
- What aspects of the patient simulation experience should we cut?
- What aspects should we emphasize and diminish?
- Are there other aspects of ED care that we should incorporate into this simulation?
- Should the length be changed? If so how?
- Should we continue to do this exercise for incoming interns? Why?
- Was this the appropriate time for trainees to do this exercise? Would it be useful for medical students or senior residents?
- Is there anything else that you would like to share?

**SUMMARY QUESTIONS**

- Having reflected today on your experience during the simulation, can you summarize how you will remember and incorporate this experience in your clinical practice going forward?

**CLOSING/WRAP UP**

(Turn to recorder). ______________________ do you have any questions? Is there anything we said that needs clarification?

OK. Thank you all very much for your participation. The information each of you have us will be very helpful to us in understanding how a patient experience simulation can be used in residency training. Do you think we missed talking about something that is important to you?

(If there is an issue, ask about it if there is time and continue the discussion. If time permits, use this opportunity to clear up any misconceptions that came out during the discussion).

I want to remind you that this discussion is confidential for everyone in this room. So, when you leave, what was said stays in this room. Thank you again for being here today.
